# Therapeutic potential of convalescent plasma and hyperimmune immunoglobulins against SARS-CoV-2 BQ.1, BQ.1.1, and XBB variants

**DOI:** 10.1172/JCI168583

**Published:** 2023-04-17

**Authors:** Lorenza Bellusci, Hana Golding, Surender Khurana

**Affiliations:** Division of Viral Products, Center for Biologics Evaluation and Research, FDA, Silver Spring, Maryland, USA.

**Keywords:** COVID-19, Therapeutics, Adaptive immunity, Immunotherapy

**To the Editor:** Convalescent plasma (CP) and hyperimmune intravenous immunoglobulins (IVIGs) are routinely used to treat patients with COVID-19. SARS-CoV-2 Omicron variants continue to evolve, generating multiple sublineages with increased transmissibility and antibody-escape mutations (1, 2). Several Omicron lineages that are currently circulating (BA.4, BA.5, BA.2.75, BA.2.75.2, BQ.1, BQ.1.1, and recombinant XBB) contain many mutations in spike protein (Supplemental Table 1; supplemental material available online with this article; https://doi.org/10.1172/JCI168583DS1), resulting in resistance to most therapeutic monoclonal antibodies as well as antibodies generated by SARS-CoV-2 vaccines (1, 2).

Hyperimmune anti–SARS-CoV-2 IVIGs (hCoV-2IG) have been manufactured from pooled plasma units of hundred to thousands of convalescent individuals. hCoV-2IG contain immunoglobulin G at a 10-fold higher concentration compared with that in CP, and they are being evaluated for treatment of COVID-19 (3).

To evaluate their therapeutic potential, 19 lots of hCoV-2IG prepared from convalescent individuals infected with SARS-CoV-2 in 2020, prior to circulation of Omicron; 20 IVIG preparations manufactured in 2019 (2019-IVIG) before the COVID-19 pandemic; and 8 IVIG lots manufactured from healthy plasma donations in 2020 (2020-IVIG) were analyzed for neutralization of SARS-CoV-2 Omicron BA.4/BA.5, BA.2.75, BA.2.75.2, BQ.1, BQ.1.1, and XBB subvariants in a pseudovirus neutralization assay (PsVNA) (Supplemental Methods). For comparison, we evaluated 8 CP samples from recovered patients with COVID-19 in early 2020 (2020-CP) and 9 CP samples from Omicron vaccine-breakthrough infections in 2022 (2022-CP).

2020-CP showed variable PsVNA50 titers against WA-1, ranging between 10 and 1,343 (geometric mean titer [GMT]: 154), but did not neutralize BQ.1, BQ.1.1, or XBB (Figure 1A and Supplemental Table 2). In contrast, 2022-CP demonstrated robust PsVNA50 titers against WA-1 (GMT: 926) and most neutralized BA.2.75, BA.2.75.2, and BA.4/BA.5 (GMT: 50, 59, and 71, respectively). However, only 4 2022-CP showed low neutralization of BQ.1 (GMT: 25), BQ.1.1 (GMT: 22), and XBB (GMT: 21).

As expected, the 2019-IVIG lots did not neutralize any SARS-CoV-2 strain. The 2020-IVIG lots (made from plasma units that were not screened for anti–SARS-CoV-2 neutralizing antibodies) had low PsVNA50 titers against WA-1 (GMT: 35) and did not neutralize Omicron variants (Figure 1A and Supplemental Table 2).

The 19 hCoV-2IG lots demonstrated robust neutralization of WA-1 (GMT: 1,615) (Figure 1A). Surprisingly, all 19 lots exhibited neutralization titers against BA.4/BA.5, ranging from 47 to 205 (GMT: 83). Importantly, 15 of the 19 hCoV-2IG lots also neutralized BA.2.75 and BA.2.75.2, with PsVNA50 titers of 22–430 (GMT: 37 and 32, respectively). At least 10 hCoV-2IG lots demonstrated presence of antibodies against BQ.1, BQ.1.1, and XBB subvariants, but the neutralization titers were further reduced (GMT: 21–25; Figure 1A and Supplemental Table 2). Strong correlations were observed between PsVNA50 titers against WA-1/2020 and BA.4/BA.5, BA.2.75, and BA.2.75.2 (*P* < 0.0001) for CP and hCoV-2IG (Figure 1B). In contrast, weak insignificant correlations were observed between PsVNA50 titers against WA-1/2020 and BQ.1, BQ.1.1, and XBB (Figure 1B).

Our study demonstrates that some hyperimmune COVID-IVIG lots manufactured in 2020 (2020-hCoV-2IG) neutralized several Omicron variants, similar to CP, from Omicron breakthrough infections in individuals with prior vaccination (2022-CP), at a level (PsVNA50 titer of >1:40) predicted to provide protection against severe COVID-19 (4). Nevertheless, evolution of the variant landscape can increase resistance to antibodies elicited by prior SARS-CoV-2 infections and vaccination, especially against the newly emerged BQ.1, BQ.1.1, and XBB subvariants (5). Therefore, high-titer hCoV-2IG batches could be generated from donors who have been boosted recently with Omicron-containing bivalent vaccine and/or recovered from infection with Omicron following vaccination (hybrid immunity) (6). While there are logistical challenges to hyperimmune globulin production (e.g., long lead time), hCoV-2IG have notable advantages over CP, including standardization of dose, pathogen reduction, and measurements of anti–SARS-CoV-2 neutralizing titers prior to release. This could improve the hCoV-2IG therapeutic effectiveness against severe COVID-19 caused by circulating and emerging SARS-CoV-2 variants.

## Author contributions

SK and HG designed research. HG provided clinical specimens and unblinded clinical data. LB and SK performed assays. SK and HG contributed to manuscript writing.

## Supplementary Material

Supplemental data

## Figures and Tables

**Figure 1:**
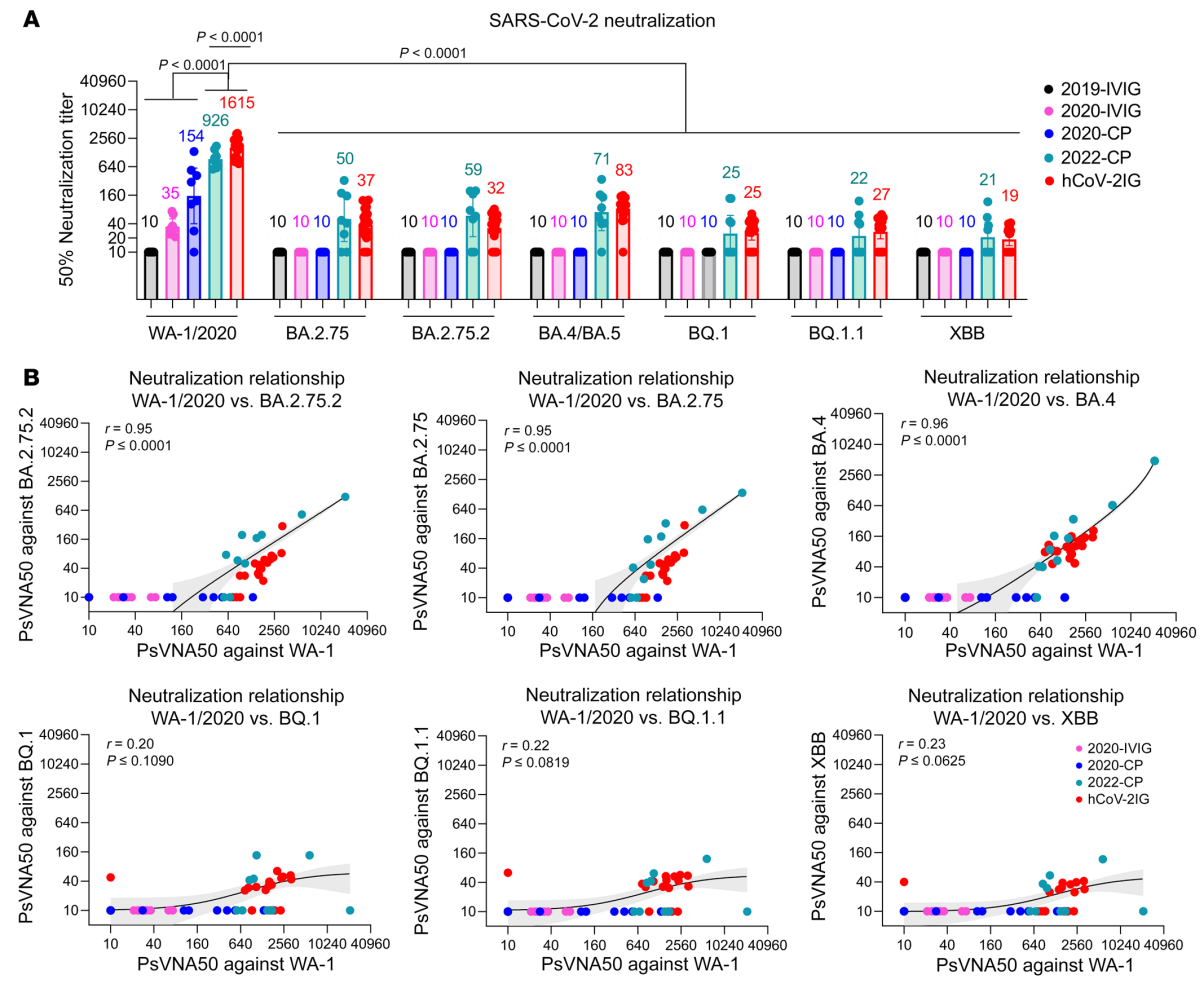
Neutralization of SARS-CoV-2 WA-1/2020 strain and Omicron subvariants by IVIG, convalescent plasma, and hCoV-2IG. (**A**) SARS-CoV-2 neutralization assays were performed using pseudoviruses expressing the spike protein of WA-1/2020 or the Omicron subvariants in 293-ACE2-TMPRSS2 cells. SARS-CoV-2 neutralization titers were determined in each of the prepandemic 2019-IVIG (*n* = 20; black), 2020-IVIG (*n* = 8; pink), 2020 convalescent plasma (2020-CP; *n* = 8; blue), 2022 convalescent plasma (2022-CP; *n* = 9; turquoise), and hCoV-2IG (*n* = 19; red) preparations. The assay was performed in duplicate to determine the 50% neutralization titer (PsVNA50). The heights of the bars and the numbers over the bars indicate the geometric mean titers, and the whiskers indicate 95% CIs. The horizontal dashed line indicates the limit of detection for the neutralization assay (PsVNA50 of 20). Differences between SARS-CoV-2 strains were analyzed by ordinary 1-way ANOVA, using Tukey’s pairwise multiple-comparison test in GraphPad Prism version 9.3.1, and *P* values are shown. (**B**) Relationship of neutralizing antibodies against SARS-CoV-2 WA-1/2020 and Omicron subvariants. Correlation of SARS-CoV-2 WA-1/2020 neutralizing titer versus Omicron subvariant neutralizing titer for 2020-CP (*n* = 8; blue), 2022-CP (*n* = 9; turquoise), and hCoV-2IG (*n* = 19; red). Correlations show Pearson’s correlation coefficient (*r*) and 2-tailed *P* values for all samples. The black lines in the scatter plots depict the linear fit of log_2_-transformed PsVNA50 values, with shaded area showing 95% CI.

## Version 1 02/23/2023

In-Press Preview

## Version 2 04/17/2023

Electronic publication
