# Supplementary material for: Therapeutic potential of convalescent plasma and hyperimmune immunoglobulins against SARS-CoV-2 BQ.1, BQ.1.1, and XBB variants
Source: J Clin Invest. 2023 Apr 17;133(8):e168583. doi: 10.1172/JCI168583 (PMC10104891; doi:10.1172/JCI168583)
Supplement: Supplemental data [file jci-133-168583-s089.pdf]

SUPPLEMENTAL APPENDIX

Supplement to:

Therapeutic potential of convalescent plasma and SARS-CoV-2 hyperimmune immunoglobulins against BQ.1, BQ.1.1 and XBB variants

by Lorenza Bellusci, Hana Golding and Surender Khurana.

TABLE OF CONTENTS

CONTENTS

1. Materials and Methods

- Samples and study design
- Ethics Statement
- Neutralization assay
- Quantification and statistical analysis

2. Table S1. SARS-CoV-2 variants mutations introduced in the spike plasmid for production of SARS-CoV-2 pseudovirions for analysis in PsVNA

3. Table S2. Summary of CP and IVIG lots and neutralization titers

4. Acknowledgments

5. Supplementary References

MATERIALS AND METHODS

Samples and study design

IVIG products approved in the United States are polyclonal antibody preparations made from 10,000 or more U.S. plasma donors and may include cold alcohol fractionation (Cohn-Oncley), anion-exchange and size-exclusion chromatographies. The final product is sterile-filtered IgG (>95%) and formulated at 100 mg/mL. Twenty intravenous immunoglobulin batches were produced from plasma collected prior to August 2019 (2019-IVIG) and eight IVIG lots made from plasma donations in 2020 (2020-IVIG) and manufactured between October 2020 and January 2021 (each lot derived from >10,000 donors), were obtained from six manufacturers.

Nineteen hCoV-2IG batches prepared from COVID-19 CP (~200-1000 donors per lot) were obtained/purchased from four commercial companies for blinded antibody analysis. The plasma units used in the manufacturing of the hCoV-2IG batches were collected in 2020 prior to emergence of the Delta and Omicron VOCs or prior to availability of COVID-19 vaccines.

Eight random CP lots were obtained from recovered COVID-19 patients between May-September 2020 (at least 30-days post-recovery) prior to COVID-19 vaccinations. At the time of collection SARS-CoV-2 D614G was the predominant strain in the US. Nine CP lots were collected in February 2022 from recovered individuals following Omicron breakthrough infections (probably BA.1), who received at least two doses of COVID-mRNA vaccination.

Ethics Statement

This study was approved by the Food and Drug Administration's Research Involving Human Subjects Committee (RIHSC #2020-04-02). This study complied with all relevant ethical

regulations for work with human participants, and informed consent was obtained. Samples were collected from adult subjects who provided informed consent to participate in the study. All assays performed fell within the permissible usages in the original informed consent.

Neutralization assay

Samples were evaluated in a qualified SARS-CoV-2 pseudovirion neutralization assay (PsVNA) using SARS-CoV-2 WA1/2020 strain and circulating Omicron subvariants: BA.4/BA.5, BA.2.75, BA.2.75.2, BQ.1, BQ.1.1 and recombinant XBB. The mutations in spike protein of these Omicron subvariants are shown in Supplementary Table S1. SARS-CoV-2 neutralizing activity measured by PsVNA correlates with PRNT (plaque reduction neutralization test with authentic SARS-CoV-2 virus) in previous studies (1-3). However, some antibodies targeting the N-terminal domain of SARS-Cov-2 spike may not show neutralization in the pseudovirus neutralization assay.

Neutralization assays were performed as previously described (2, 4). Briefly, 50 µL of SARS-CoV-2 S pseudovirions (counting ~200,000 relative light units) were pre-incubated with an equal volume of medium containing serial dilutions (starting at 1:10) of all samples at room temperature for 1 h. Then, 50 µL of virus-antibody mixtures were added to 293T-ACE2-TMPRSS2 cells [10^4 cells/50 µL; gift from Carol Weiss (1)] in a 96-well plate. The input virus with all SARS-CoV-2 strains was the same (2 x 10^5 relative light units/50 µL/well). After a 3 h incubation, fresh medium was added to the wells. Cells were lysed 24 h later, and luciferase activity was measured using One-Glo luciferase assay system (Promega). The assay of each sample was performed in duplicate, and the 50% neutralization titer was calculated using Prism 9 (GraphPad Software). The limit of detection for the neutralization assay is 1:20. Two

independent biological replicate experiments were performed for each sample and variation in PsVNA50 titers was <10% between replicates.

Quantification and statistical analysis

Descriptive statistics were performed to determine the geometric mean titer values and were calculated using GraphPad. All experimental data to compare differences among groups were analyzed using Ordinary one-way ANOVA with Tukey's pairwise multiple comparison test in GraphPad Prism version 9.3.1. To ensure robustness of the results, absolute measurements were log2-transformed before performing the analysis. Correlation and regression analyses were performed by computing Pearson correlation (r), assuming that the data were sampled from Gaussian Distribution, in GraphPad Prism version 9.3.1, to correlate the neutralization titers of WA-1 strain with each Omicron subvariant: BA.2.75.2, BA.2.75, BA.4, BQ.1, BQ.1.1 and XBB, for all the SARS-CoV-2 positive CP and IVIG samples. For each graph, we show the value of r (not R square) and a confidence interval of 95%, with two-tailed significance (p) values.

Data sharing. All data needed to evaluate the conclusions in the article are present in the manuscript.

Supplementary Table S1: SARS-CoV-2 variants mutations introduced in the spike plasmid for production of SARS-CoV-2 pseudovirions for analysis in PsVNA.

SARS-CoV-2 variant	Mutations constructed in the spike plasmids
**Omicron (BA.2.75)**	BA.2 spike mutations (T19I, delL24, delP25, delP26, A27S, G142D, V213G, G339D, S371F, S373P, S375F, T376A, D405N, R408S, K417N, N440K, S477N, T478K, E484A, Q493R, Q498R, N501Y, Y505H, D614G, H655Y, N679K, P681H, N764K, D796Y, Q954H, N969K) + K147E, W152R, F157L, I210V, G257S, D339H, G446S, N460K, Q493reversion
**Omicron (BA.2.75.2)**	BA.2 spike mutations (T19I, delL24, delP25, delP26, A27S, G142D, V213G, G339D, S371F, S373P, S375F, T376A, D405N, R408S, K417N, N440K, S477N, T478K, E484A, Q493R, Q498R, N501Y, Y505H, D614G, H655Y, N679K, P681H, N764K, D796Y, Q954H, N969K) + K147E, W152R, F157L, I210V, G257S, D339H, R346T, G446S, N460K, F486S, Q493reversion, D1199N
**Omicron (BA.4/BA.5)**	T19I, delL24, delP25, delP26, A27S, del69 70, G142D, V213G, G339D, S371F, S373P, S375F, T376A, D405N, R408S, K417N, N440K, L452R, S477N, T478K, E484A, F486V, Q498R, N501Y, Y505H, D614G, H655Y, N679K, P681H, N764K, D796Y, Q954H, N969K
**Omicron (BQ.1)**	BA.4/BA.5 spike mutations (T19I, delL24, delP25, delP26, A27S, del69 70, G142D, V213G, G339D, S371F, S373P, S375F, T376A, D405N, R408S, K417N, N440K, L452R, S477N, T478K, E484A, F486V, Q498R, N501Y, Y505H, D614G, H655Y, N679K, P681H, N764K, D796Y, Q954H, N969K) + K444T and N460K.
**Omicron (BQ.1.1)**	BQ.1 spike mutations (T19I, delL24, delP25, delP26, A27S, del69 70, G142D, V213G, G339D, S371F, S373P, S375F, T376A, D405N, R408S, K417N, N440K, L452R, S477N, T478K, E484A, F486V, Q498R, N501Y, Y505H, D614G, H655Y, N679K, P681H, N764K, D796Y, Q954H, N969K, K444T and N460K) + R346T.
**Omicron (XBB)**	BA.2 spike mutations (T19I, delL24, delP25, delP26, A27S, G142D, V213G, G339D, S371F, S373P, S375F, T376A, D405N, R408S, K417N, N440K, S477N, T478K, E484A, Q493R, Q498R, N501Y, Y505H, D614G, H655Y, N679K, P681H, N764K, D796Y, Q954H, N969K) + V83A, Del144, H146Q, Q183E, V213E, G339H, R346T, L368I, V445P, G446S, N460K, F486S, F490S, R493Q

Table S2: Neutralization titers of convalescent plasma, IVIG and hCoV-2IG against SARS-CoV-2 variants*

	WA-1	Omicron BA.2.75	Omicron BA.2.75.2	Omicron BA.4/BA.5	Omicron BQ.1	Omicron BQ.1.1	Omicron XBB
**IVIG batches produced in 2019 prior to COVID-19**							
**2019-IVIG-1**	10	10	10	10	10	10	10
**2019-IVIG-2**	10	10	10	10	10	10	10
**2019-IVIG-3**	10	10	10	10	10	10	10
**2019-IVIG-4**	10	10	10	10	10	10	10
**2019-IVIG-5**	10	10	10	10	10	10	10
**2019-IVIG-6**	10	10	10	10	10	10	10
**2019-IVIG-7**	10	10	10	10	10	10	10
**2019-IVIG-8**	10	10	10	10	10	10	10
**2019-IVIG-9**	10	10	10	10	10	10	10
**2019-IVIG-10**	10	10	10	10	10	10	10
**2019-IVIG-11**	10	10	10	10	10	10	10
**2019-IVIG-12**	10	10	10	10	10	10	10
**2019-IVIG-13**	10	10	10	10	10	10	10
**2019-IVIG-14**	10	10	10	10	10	10	10
**2019-IVIG-15**	10	10	10	10	10	10	10
**2019-IVIG-16**	10	10	10	10	10	10	10
**2019-IVIG-17**	10	10	10	10	10	10	10
**2019-IVIG-18**	10	10	10	10	10	10	10
**2019-IVIG-19**	10	10	10	10	10	10	10
**2019-IVIG-20**	10	10	10	10	10	10	10
**IVIG batches produced in 2020**							
**2020-IVIG-1**	31	10	10	10	10	10	10
**2020-IVIG-2**	25	10	10	10	10	10	10
**2020-IVIG-3**	73	10	10	10	10	10	10
**2020-IVIG-4**	36	10	10	10	10	10	10
**2020-IVIG-5**	64	10	10	10	10	10	10
**2020-IVIG-6**	32	10	10	10	10	10	10
**2020-IVIG-7**	24	10	10	10	10	10	10
**2020-IVIG-8**	21	10	10	10	10	10	10
**Convalescent plasma batches produced from COVID-19 survivors collected in 2020**							
**2020-CP-1**	538	10	10	10	10	10	10
**2020-CP-2**	415	10	10	10	10	10	10
**2020-CP-3**	10	10	10	10	10	10	10
**2020-CP-4**	302	10	10	10	10	10	10
**2020-CP-5**	28	10	10	10	10	10	10
**2020-CP-6**	104	10	10	10	10	10	10
**2020-CP-7**	122	10	10	10	10	10	10
**2020-CP-8**	1343	10	79	95	10	10	10
**Convalescent plasma batches produced from COVID-19 survivors collected in 2022**							
**2022-CP-1**	1748	327	198	345	136	121	116
**2022-CP-2**	1067	47	50	53	10	10	10
**2022-CP-3**	21306	1373	1214	4841	136	61	54
**2022-CP-4**	567	10	10	10	10	10	10
**2022-CP-5**	677	10	10	40	10	10	10
**2022-CP-6**	854	24	58	87	10	10	10
**2022-CP-7**	973	155	196	163	42	39	37
**2022-CP-8**	1505	176	169	145	45	42	30
**2022-CP-9**	603	41	76	41	10	10	10
**hCoV-2IG batches produced from COVID-19 convalescent plasma donors**							
**hCoV-2IG-1**	2067	43	52	128	64	53	36
**hCoV-2IG-2**	1664	49	39	71	10	10	10
**hCoV-2IG-3**	1602	27	29	81	39	44	39
**hCoV-2IG-4**	1833	10	22	47	10	10	10
**hCoV-2IG-5**	2302	126	62	103	10	10	10
**hCoV-2IG-6**	920	10	10	46	10	10	10
**hCoV-2IG-7**	3243	430	297	205	48	63	40
**hCoV-2IG-8**	1730	55	39	99	44	33	28
**hCoV-2IG-9**	819	20	10	107	34	31	10
**hCoV-2IG-10**	1428	46	50	99	29	32	10
**hCoV-2IG-11**	737	23	10	78	26	32	28
**hCoV-2IG-12**	917	24	28	85	26	37	10
**hCoV-2IG-13**	1055	64	28	81	10	10	10
**hCoV-2IG-14**	1896	63	60	104	30	42	25
**hCoV-2IG-15**	1565	36	31	59	10	10	10
**hCoV-2IG-16**	2351	84	73	143	32	54	31
**hCoV-2IG-17**	2486	90	67	151	46	43	25
**hCoV-2IG-18**	3162	124	82	154	49	57	39
**hCoV-2IG-19**	1646	29	47	159	53	55	42

* PsVNA titer Cut-off value: 1:10.

Acknowledgments

We thank Basil Golding and Keith Peden at FDA for review of the manuscript. We thank Carol Weiss (FDA) for providing plasmid clones expressing SARS-CoV-2 spike variants.

Supplementary References

1. Neerukonda SN, Vassell R, Herrup R, Liu S, Wang T, Takeda K, et al. Establishment of a well-characterized SARS-CoV-2 lentiviral pseudovirus neutralization assay using 293T cells with stable expression of ACE2 and TMPRSS2. *PLoS One.* 2021;16(3):e0248348.
2. Tang J, Lee Y, Ravichandran S, Grubbs G, Huang C, Stauft CB, et al. Epitope diversity of SARS-CoV-2 hyperimmune intravenous human immunoglobulins and neutralization of variants of concern. *iScience.* 2021:103006.
3. Ravichandran S, Coyle EM, Klenow L, Tang J, Grubbs G, Liu S, et al. Antibody signature induced by SARS-CoV-2 spike protein immunogens in rabbits. *Sci Transl Med.* 2020;12(550):eabc3539.
4. Ravichandran S, Lee Y, Grubbs G, Coyle EM, Klenow L, Akasaka O, et al. Longitudinal antibody repertoire in "mild" versus "severe" COVID-19 patients reveals immune markers associated with disease severity and resolution. *Sci Adv.* 2021;7(10).
